# Texas “Sanitation” by Force and Arms

**Published:** 1899-04

**Authors:** 


### Editorial Department.

F. E. DANIEL, M. D., Editor.
S. E. HUDSON, M. D., Managing Editor.
A. J. SMITH. M. D., Galveston, Associate Editor.

Published Monthly at Austin, Texas, by Drs. Daniel and Hudson. Subscription price $1.00 a year in advance.

Eastern Representative: John Guy Monihan, St. Paul Building, 220 Broadway, New York City.

Official organ of the West Texas Medical Association, the Houston District Medical Association, the Austin District Medical Society, the Brazos Valley Medical Association, the Galveston County Medical Society, and several others.

### Texas "Sanitation" by Force and Arms.

Mark Twain, in his inimitable story, "A Nevada Funeral," tells of the arrangements made to give the "late lamented" Buck Fanshaw a "good send off." One Scotty Briggs, closest friend and chief mourner, went in search of a minister of the gospel. He had some difficulty in making the local preacher, a newly arrived little "pale theological student from Boston," understand his wishes when he inquired: "Are you the duck that runs the doxology works?" but finally succeeded when he said that he was in search of "a gospel sharp to chuck chin music" over the remains of a friend,—"the whitest man that ever lived," a man who "never shook his mother." Catching on bye and bye, the little preacher began to question Scotty as to the facts in the life of deçeased, so as to prepare a suitable tribute to his memory; and amongst other things, he asked: "Was your friend, the late Mr. Fanshaw, a peaceable man?"

"Peaceable!" exclaimed Scotty, "he was the damndest peaceablest man you ever *seen!* He would *have* peace. Why, I saw him lick eleven 'greasers' in fifteen minutes because they wouldn't be peaceable!"

(Uncle Sam seems to be of Scotty's way of thinking, with regard to the Philippinos; but "that's another story.")

The wherefore of the foregoing remarks is to be found in the recent "stamping out" (?) of the smallpox epidemic in Laredo—*vi et armis;* "sanitation" with gatling-gun adjunct.

Under the law in Texas, each county is required to deal with local epidemics, and to pay the expense. I will remark here, incidentally, that in 1895 one case of smallpox cost Nacogdoches county nine hundred and fifty dollars, and a small outbreak in Navarro county (I think it was Navarro) cost the county over eighteen hundred dollars. (See Swearingen's report for that year.) The law provides, however, that in case of failure or refusal of the county authorities to take hold of the matter, supposing an outbreak of smallpox or other epidemic disease to occur, the State shall assume charge; but I do not know of any warrant in law for removing smallpox patients from their own homes to a pest house in opposition to the wishes of the family, and certainly none for doing so at the mouth of a gun in the hands of United States cavalrymen, backed by a gatling-gun in position. This was done at Laredo. Nor do I know of any warrant in law for burning an infected house, as was done in Dallas some years ago, and no remuneration to the owner.

Laredo, as everybody knows, is on the Rio Grande, at the crossing of the Texas-Mexican Railroad. The Rio Grande is the boundary between Texas and Mexico for several hundred miles. It is the policy of the State to keep an inspector on duty all the year round at each of the three railroad crossings, Laredo, El Paso and Eagle Pass. These gentlemen get, each, $1800 a year. They have nothing to do with local epidemics; they only mind the gap. The other expense of keeping up these stations varies, and cuts no particular figure here. I am aiming only to show the want of wisdom displayed in eternally dealing with effects, and ignoring *causes;* and that a wiser course could be pursued whereby this absurd situation would not be necessary. The river is fordable at almost any point for five hundred miles the greater part of each year. It would require a standing army, say a thousand guards, to successfully guard the border against the crossing of Mexicans into Texas, and the State's policy of minding the three railroad crossings only, is like bolting the door of one's own house and leaving the windows wide open. Smallpox gained such headway in Laredo that the citizens called on the State to take charge. Mr. Sayers asked the Legislature, now in session, to give the State Health Officer $2000—outside of the regular appropriation—with which to "handle the case," and they promptly did so. The State Health Officer thought it advisable to concentrate the sick and (of course) to vaccinate those who had been exposed, but when he attempted to do this, to remove the patients to the pest house, the Mexicans *resisted.* The State Health Officer then called for a detachment of State Rangers—

mounted State police—which was promptly sent him, but the Mexicans shot the captain through the shoulder, knocking the Rangers out. A *squad of United States negro cavalry with a gatling-gun* was then sent to the doctor from Fort Davis, and the "mob" was dispersed (*only* some three or four were killed), and the patients were all taken to the pest house, and all is (reported to be) "lovely" —as far as heard from. The Marine Hospital Service Reports put the mortality from the disease at Laredo, from December 31 to March 3, 1899, at 36 per cent. (72 deaths; 200 cases). We have no report later of the mortality, but at this writing, April 6, there are 175 cases at the pest house.

It is not my intention by any means to reflect on the State Health Officer. He is a bonded officer, and sworn to execute the law. If the law is bad, it is not his fault, and he is not responsible for it, and it surely is inefficient in many particulars; were it not, the assistance of the United States Army would not be needed; but I raise the question: was Dr. Blunt executing the law in this instance? Where, in the law, is any warrant for forcibly taking an inmate of a man's house to the pest house? If the law warrants such proceeding, and the State is unable to enforce it without the assistance of the United States Army, the sooner that law is repealed, or the sooner the State surrenders all its police powers to the Federal government, the better. If there is authority in law for such proceedings—and I deny it—no man's home is safe from such invasion. But—has a *Mexican* any rights which a Texas Governor or State Health Officer is bound to respect? A law that applies to a Mexican citizen should apply to an American citizen. If, by any chance, the conditions Dr. Blunt found existing at Laredo amongst these humble, ignorant and superstitious *citizens* (are they "citizens" of the United States,—or, aliens, sojourning temporarily on our shores? If the latter, it is a matter for *international* adjustment) should obtain at San Antonio or Austin or other city; if the *better class* should be the victims of smallpox,—would the State Health Officer dare—would the Governor—venture to cause the removal of the sick by force from their homes to a pest house?—Would he dare to so take my child, or yours, to a pest house?

We ask readers of the above to please read elsewhere in this issue the account by Dr. Kenney of his management of a smallpox epidemic is near-by New Mexico. He treated cases where he found them; removed none to a pest house, had no $2000, and had no need of soldiers to vaccinate the well with buckshot, nor to assist him in vaccinating with lymph. He did *burn a house,* however; but that was not in Texas.

Existing conditions render a recurrence of epidemics of smallpox in Texas imminent at all times. The disease is endemic in Mexico the year around. The great mass of Mexicans—priest ridden for centuries—are opposed to vaccination; they cling to the old superstitious belief that smallpox is a "visitation" sent upon them for their sins, and that it is sinful to try to prevent it. Dr. Yandell, quarantine officer at El Paso, who has been much among the Mexican laboring and vagabond classes, informs me that families will carry their children miles purposely to expose them to smallpox, that they may "have it and be done with it." If they survive, they are safe in after life, and if they die, it is the "will of God." The river being fordable almost anywhere, there is always a free commingling of these classes with their kind on the Texas side; hence, so long as Texas "sanitation" is content to deal with effects, and to ignore causes, so long will this great danger and expense continue.

Smallpox is preventable. No fact in medical science is more firmly established; no fact has ever been more clearly and oftener demonstrated than that vaccination will prevent smallpox. I am under the impression, in fact, I am quite sure the United States immigration laws prohibit the immigration of foreigners who are not protected by vaccination; and no immigrant is permitted to land, at Castle Garden, at least, who has not been vaccinated; if such should arrive, the port health officer promptly vaccinates him. Why not apply this rule to Mexicans?

In short, would it not be wiser,—more rational, more economical, more humane,—more in keeping with an (alleged) advanced civilization, to *make vaccination compulsory* throughout Texas—make it a condition to even temporary residence in any part of the State, than to be constantly exposed to such danger of outbreaks of the disease as exists at present, and to be forced to resort to such brutal and barbarous methods of dealing with smallpox as those practiced at Laredo?

We surely are a primitive people in Texas.

